# Variables Impacting Nursing Home Goals of Care Discussions and Order Implementation During COVID-19

**DOI:** 10.1093/geroni/igab046.2716

**Published:** 2021-12-17

**Authors:** Orah Burack, Joann Reinhardt, Wingyun Mak, Himali Weerahandi, Benjamin Canter, Kenneth Boockvar

**Affiliations:** 1 The New Jewish Home, New York, New York, United States; 2 NYU Grossman School of Medicine, New York, New York, United States; 3 The New Jewish Home, The New Jewish Home, New York, United States

## Abstract

Nursing home (NH) residents are especially vulnerable to COVID-19, disproportionately suffering from severe illness and death. As such, resident Goals of Care (GOC) often had to be quickly established to ensure treatment preferences were known and respected. This study examined variables related to the occurrence of GOC discussions and added orders (Do Not Resuscitate, Do Not Intubate, and Do Not Hospitalize), including demographic, physical functioning, cognitive impairment, depression, number of diagnoses, and Optum participation (Optum provided added specialized care by nurse practitioners who routinely address GOC preferences). Subjects were 286 COVID positive residents from a large NYC NH. All data were obtained from the NH’s electronic medical records. Patient median age was 81 n (interquartile range 71-88), 59% were female, 61% were long stay (stay >100 days) and 39% were short stay. Using bivariate correlations we found that older short stay residents were more likely to have GOC conversations. Additionally, older, cognitively impaired, Optum participants were more likely to have orders added. When all independent variables were entered into binary logistic regressions, only older age and being a primary English speaker were significantly related to the occurrence of GOC conversations (□2= 21.76**; N=278; Nagelkerke R2 = .10), while older age and being an Optum participant were related to added orders (□2=32.18**; N=164; Nagelkerke R2 = .24). Results have implications for (1) ensuring the GOC wishes of diverse populations are known and abided by and (2) improving the quality of clinician – resident GOC discussions.

